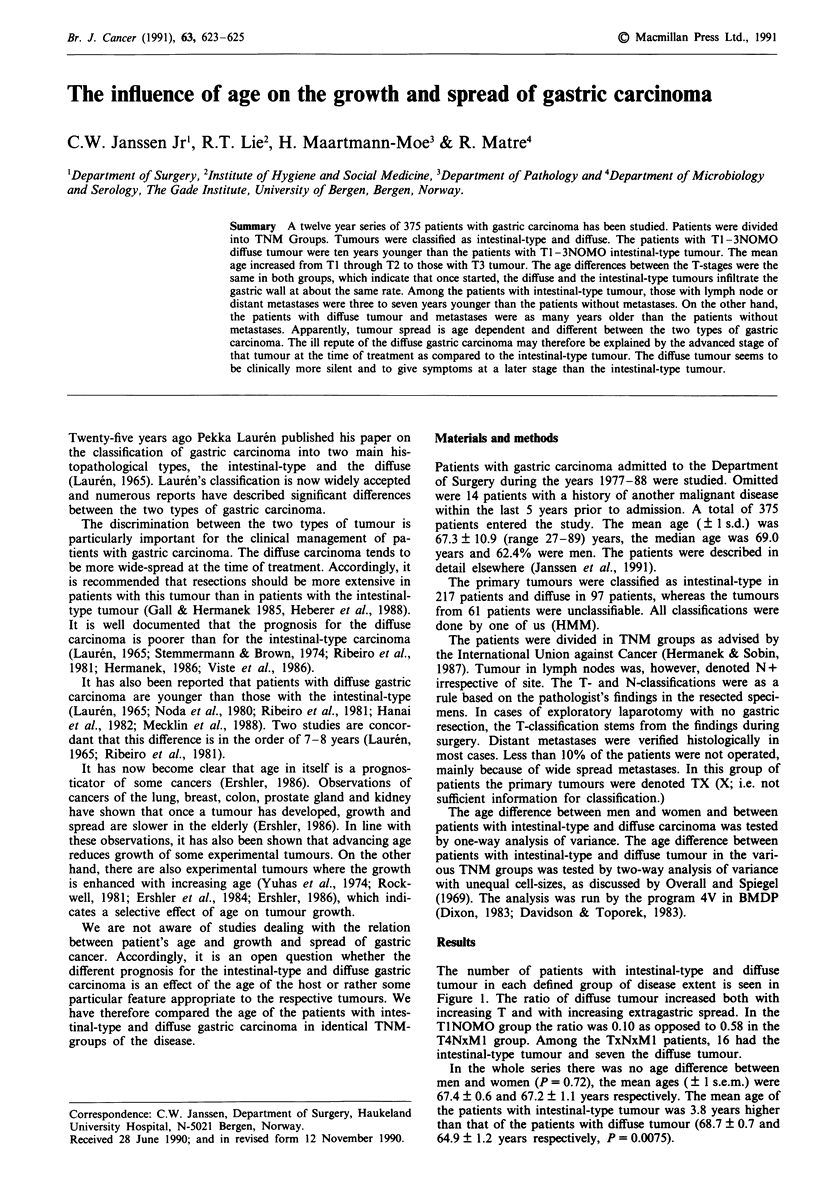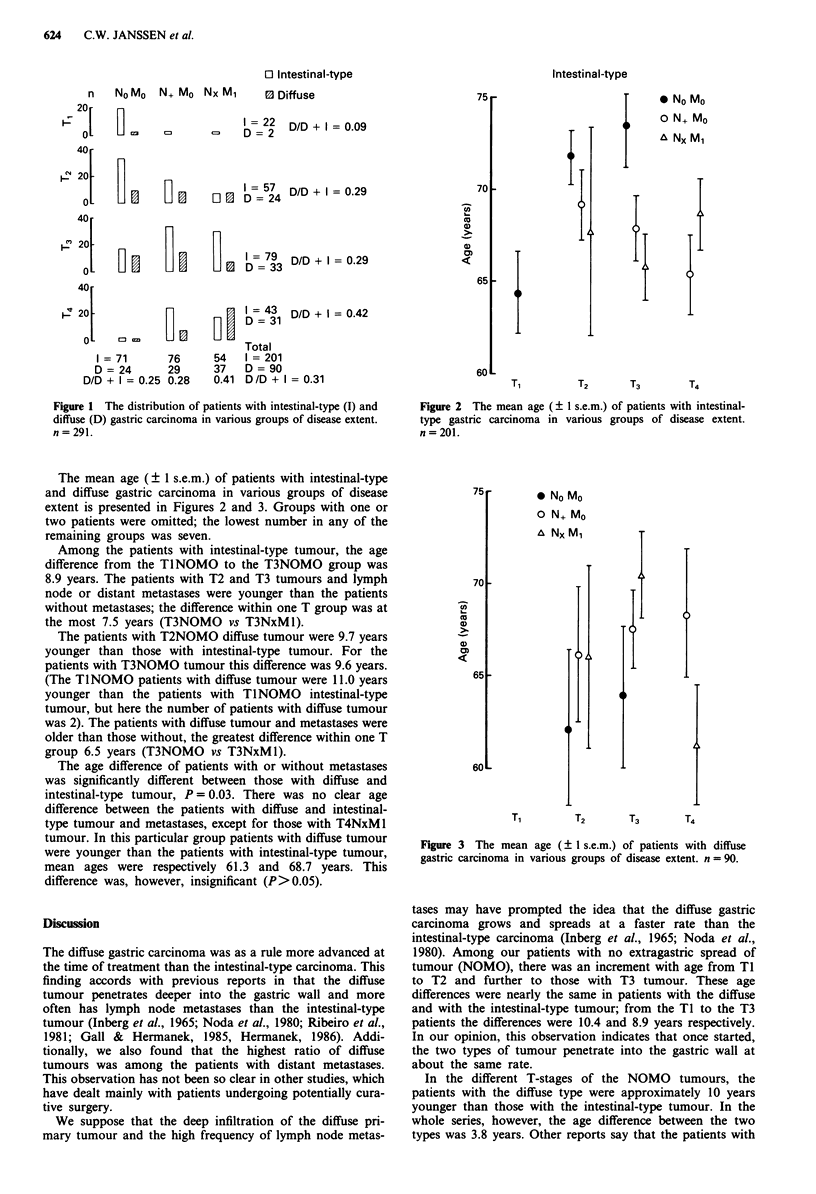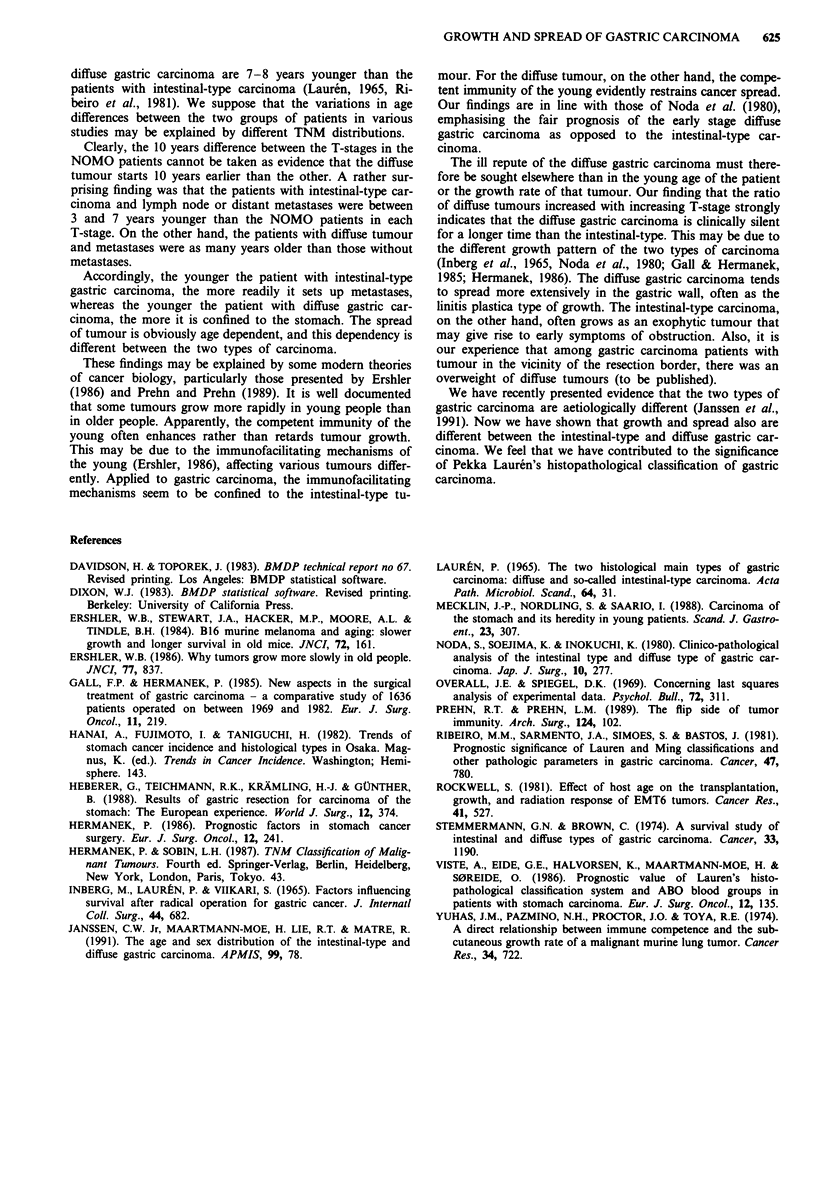# The influence of age on the growth and spread of gastric carcinoma.

**DOI:** 10.1038/bjc.1991.142

**Published:** 1991-04

**Authors:** C. W. Janssen, R. T. Lie, H. Maartmann-Moe, R. Matre

**Affiliations:** Department of Surgery, Gade Institute, University of Bergen, Norway.

## Abstract

A twelve year series of 375 patients with gastric carcinoma has been studied. Patients were divided into TNM Groups. Tumours were classified as intestinal-type and diffuse. The patients with T1-3NOMO diffuse tumour were ten years younger than the patients with T1-3NOMO intestinal-type tumour. The mean age increased from T1 through T2 to those with T3 tumour. The age differences between the T-stages were the same in both groups, which indicate that once started, the diffuse and the intestinal-type tumours infiltrate the gastric wall at about the same rate. Among the patients with intestinal-type tumour, those with lymph node or distant metastases were three to seven years younger than the patients without metastases. On the other hand, the patients with diffuse tumour and metastases were as many years older than the patients without metastases. Apparently, tumour spread is age dependent and different between the two types of gastric carcinoma. The ill repute of the diffuse gastric carcinoma may therefore be explained by the advanced stage of that tumour at the time of treatment as compared to the intestinal-type tumour. The diffuse tumour seems to be clinically more silent and to give symptoms at a later stage than the intestinal-type tumour.


Br. J. Cancer (1991), 63, 623-625 © Macmillan Press Ltd., 1991

### The influence of age on the growth and spread of gastric carcinoma

C.W. Janssen Jr[1], R.T. Lie[2], H. Maartmann-Moe[3] & R. Matre[4]

[1]*Department of Surgery,* [2]*Institute of Hygiene and Social Medicine,* [3]*Department of Pathology and* [4]*Department of Microbiology and Serology, The Gade Institute, University of Bergen, Bergen, Norway.*

**Summary** A twelve year series of 375 patients with gastric carcinoma has been studied. Patients were divided into TNM Groups. Tumours were classified as intestinal-type and diffuse. The patients with T1-3NOMO diffuse tumour were ten years younger than the patients with T1-3NOMO intestinal-type tumour. The mean age increased from T1 through T2 to those with T3 tumour. The age differences between the T-stages were the same in both groups, which indicate that once started, the diffuse and the intestinal-type tumours infiltrate the gastric wall at about the same rate. Among the patients with intestinal-type tumour, those with lymph node or distant metastases were three to seven years younger than the patients without metastases. On the other hand, the patients with diffuse tumour and metastases were as many years older than the patients without metastases. Apparently, tumour spread is age dependent and different between the two types of gastric carcinoma. The ill repute of the diffuse gastric carcinoma may therefore be explained by the advanced stage of that tumour at the time of treatment as compared to the intestinal-type tumour. The diffuse tumour seems to be clinically more silent and to give symptoms at a later stage than the intestinal-type tumour.

Twenty-five years ago Pekka Laurén published his paper on the classification of gastric carcinoma into two main histopathological types, the intestinal-type and the diffuse (Laurén, 1965). Laurén's classification is now widely accepted and numerous reports have described significant differences between the two types of gastric carcinoma.

The discrimination between the two types of tumour is particularly important for the clinical management of patients with gastric carcinoma. The diffuse carcinoma tends to be more wide-spread at the time of treatment. Accordingly, it is recommended that resections should be more extensive in patients with this tumour than in patients with the intestinal-type tumour (Gall & Hermanek 1985, Heberer *et al.*, 1988). It is well documented that the prognosis for the diffuse carcinoma is poorer than for the intestinal-type carcinoma (Laurén, 1965; Stemmermann & Brown, 1974; Ribeiro *et al.*, 1981; Hermanek, 1986; Viste *et al.*, 1986).

It has also been reported that patients with diffuse gastric carcinoma are younger than those with the intestinal-type (Laurén, 1965; Noda *et al.*, 1980; Ribeiro *et al.*, 1981; Hanai *et al.*, 1982; Mecklin *et al.*, 1988). Two studies are concordant that this difference is in the order of 7-8 years (Laurén, 1965; Ribeiro *et al.*, 1981).

It has now become clear that age in itself is a prognosticator of some cancers (Ershler, 1986). Observations of cancers of the lung, breast, colon, prostate gland and kidney have shown that once a tumour has developed, growth and spread are slower in the elderly (Ershler, 1986). In line with these observations, it has also been shown that advancing age reduces growth of some experimental tumours. On the other hand, there are also experimental tumours where the growth is enhanced with increasing age (Yuhas *et al.*, 1974; Rockwell, 1981; Ershler *et al.*, 1984; Ershler, 1986), which indicates a selective effect of age on tumour growth.

We are not aware of studies dealing with the relation between patient's age and growth and spread of gastric cancer. Accordingly, it is an open question whether the different prognosis for the intestinal-type and diffuse gastric carcinoma is an effect of the age of the host or rather some particular feature appropriate to the respective tumours. We have therefore compared the age of the patients with intestinal-type and diffuse gastric carcinoma in identical TNM-groups of the disease.

Correspondence: C.W. Janssen, Department of Surgery, Haukeland University Hospital, N-5021 Bergen, Norway.
Received 28 June 1990; and in revised form 12 November 1990.

### Materials and methods

Patients with gastric carcinoma admitted to the Department of Surgery during the years 1977-88 were studied. Omitted were 14 patients with a history of another malignant disease within the last 5 years prior to admission. A total of 375 patients entered the study. The mean age (± 1 s.d.) was 67.3 ± 10.9 (range 27-89) years, the median age was 69.0 years and 62.4% were men. The patients were described in detail elsewhere (Janssen *et al.*, 1991).

The primary tumours were classified as intestinal-type in 217 patients and diffuse in 97 patients, whereas the tumours from 61 patients were unclassifiable. All classifications were done by one of us (HMM).

The patients were divided in TNM groups as advised by the International Union against Cancer (Hermanek & Sobin, 1987). Tumour in lymph nodes was, however, denoted N+ irrespective of site. The T- and N-classifications were as a rule based on the pathologist's findings in the resected specimens. In cases of exploratory laparotomy with no gastric resection, the T-classification stems from the findings during surgery. Distant metastases were verified histologically in most cases. Less than 10% of the patients were not operated, mainly because of wide spread metastases. In this group of patients the primary tumours were denoted TX (X; i.e. not sufficient information for classification.)

The age difference between men and women and between patients with intestinal-type and diffuse carcinoma was tested by one-way analysis of variance. The age difference between patients with intestinal-type and diffuse tumour in the various TNM groups was tested by two-way analysis of variance with unequal cell-sizes, as discussed by Overall and Spiegel (1969). The analysis was run by the program 4V in BMDP (Dixon, 1983; Davidson & Toporek, 1983).

### Results

The number of patients with intestinal-type and diffuse tumour in each defined group of disease extent is seen in Figure 1. The ratio of diffuse tumour increased both with increasing T and with increasing extragastric spread. In the T1NOMO group the ratio was 0.10 as opposed to 0.58 in the T4NxM1 group. Among the TxNxM1 patients, 16 had the intestinal-type tumour and seven the diffuse tumour.

In the whole series there was no age difference between men and women ($P = 0.72$), the mean ages (± 1 s.e.m.) were 67.4 ± 0.6 and 67.2 ± 1.1 years respectively. The mean age of the patients with intestinal-type tumour was 3.8 years higher than that of the patients with diffuse tumour (68.7 ± 0.7 and 64.9 ± 1.2 years respectively, $P = 0.0075$).

**Figure 1** The distribution of patients with intestinal-type (I) and diffuse (D) gastric carcinoma in various groups of disease extent. $n = 291$.

**Figure 2** The mean age ($\pm$ 1 s.e.m.) of patients with intestinal-type gastric carcinoma in various groups of disease extent. $n = 201$.

The mean age ($\pm$ 1 s.e.m.) of patients with intestinal-type and diffuse gastric carcinoma in various groups of disease extent is presented in Figures 2 and 3. Groups with one or two patients were omitted; the lowest number in any of the remaining groups was seven.

Among the patients with intestinal-type tumour, the age difference from the T1NOMO to the T3NOMO group was 8.9 years. The patients with T2 and T3 tumours and lymph node or distant metastases were younger than the patients without metastases; the difference within one T group was at the most 7.5 years (T3NOMO *vs* T3NxM1).

The patients with T2NOMO diffuse tumour were 9.7 years younger than those with intestinal-type tumour. For the patients with T3NOMO tumour this difference was 9.6 years. (The T1NOMO patients with diffuse tumour were 11.0 years younger than the patients with T1NOMO intestinal-type tumour, but here the number of patients with diffuse tumour was 2). The patients with diffuse tumour and metastases were older than those without, the greatest difference within one T group 6.5 years (T3NOMO *vs* T3NxM1).

The age difference of patients with or without metastases was significantly different between those with diffuse and intestinal-type tumour, $P = 0.03$. There was no clear age difference between the patients with diffuse and intestinal-type tumour and metastases, except for those with T4NxM1 tumour. In this particular group patients with diffuse tumour were younger than the patients with intestinal-type tumour, mean ages were respectively 61.3 and 68.7 years. This difference was, however, insignificant ($P > 0.05$).

**Figure 3** The mean age ($\pm$ 1 s.e.m.) of patients with diffuse gastric carcinoma in various groups of disease extent. $n = 90$.

### Discussion

The diffuse gastric carcinoma was as a rule more advanced at the time of treatment than the intestinal-type carcinoma. This finding accords with previous reports in that the diffuse tumour penetrates deeper into the gastric wall and more often has lymph node metastases than the intestinal-type tumour (Inberg *et al.*, 1965; Noda *et al.*, 1980; Ribeiro *et al.*, 1981; Gall & Hermanek, 1985, Hermanek, 1986). Additionally, we also found that the highest ratio of diffuse tumours was among the patients with distant metastases. This observation has not been so clear in other studies, which have dealt mainly with patients undergoing potentially curative surgery.

We suppose that the deep infiltration of the diffuse primary tumour and the high frequency of lymph node metastases may have prompted the idea that the diffuse gastric carcinoma grows and spreads at a faster rate than the intestinal-type carcinoma (Inberg *et al.*, 1965; Noda *et al.*, 1980). Among our patients with no extragastric spread of tumour (NOMO), there was an increment with age from T1 to T2 and further to those with T3 tumour. These age differences were nearly the same in patients with the diffuse and with the intestinal-type tumour; from the T1 to the T3 patients the differences were 10.4 and 8.9 years respectively. In our opinion, this observation indicates that once started, the two types of tumour penetrate into the gastric wall at about the same rate.

In the different T-stages of the NOMO tumours, the patients with the diffuse type were approximately 10 years younger than those with the intestinal-type tumour. In the whole series, however, the age difference between the two types was 3.8 years. Other reports say that the patients with diffuse gastric carcinoma are 7–8 years younger than the patients with intestinal-type carcinoma (Laurén, 1965, Ribeiro *et al.*, 1981). We suppose that the variations in age differences between the two groups of patients in various studies may be explained by different TNM distributions.

Clearly, the 10 years difference between the T-stages in the NOMO patients cannot be taken as evidence that the diffuse tumour starts 10 years earlier than the other. A rather surprising finding was that the patients with intestinal-type carcinoma and lymph node or distant metastases were between 3 and 7 years younger than the NOMO patients in each T-stage. On the other hand, the patients with diffuse tumour and metastases were as many years older than those without metastases.

Accordingly, the younger the patient with intestinal-type gastric carcinoma, the more readily it sets up metastases, whereas the younger the patient with diffuse gastric carcinoma, the more it is confined to the stomach. The spread of tumour is obviously age dependent, and this dependency is different between the two types of carcinoma.

These findings may be explained by some modern theories of cancer biology, particularly those presented by Ershler (1986) and Prehn and Prehn (1989). It is well documented that some tumours grow more rapidly in young people than in older people. Apparently, the competent immunity of the young often enhances rather than retards tumour growth. This may be due to the immunofacilitating mechanisms of the young (Ershler, 1986), affecting various tumours differently. Applied to gastric carcinoma, the immunofacilitating mechanisms seem to be confined to the intestinal-type tumour. For the diffuse tumour, on the other hand, the competent immunity of the young evidently restrains cancer spread. Our findings are in line with those of Noda *et al.* (1980), emphasising the fair prognosis of the early stage diffuse gastric carcinoma as opposed to the intestinal-type carcinoma.

The ill repute of the diffuse gastric carcinoma must therefore be sought elsewhere than in the young age of the patient or the growth rate of that tumour. Our finding that the ratio of diffuse tumours increased with increasing T-stage strongly indicates that the diffuse gastric carcinoma is clinically silent for a longer time than the intestinal-type. This may be due to the different growth pattern of the two types of carcinoma (Inberg *et al.*, 1965, Noda *et al.*, 1980; Gall & Hermanek, 1985; Hermanek, 1986). The diffuse gastric carcinoma tends to spread more extensively in the gastric wall, often as the linitis plastica type of growth. The intestinal-type carcinoma, on the other hand, often grows as an exophytic tumour that may give rise to early symptoms of obstruction. Also, it is our experience that among gastric carcinoma patients with tumour in the vicinity of the resection border, there was an overweight of diffuse tumours (to be published).

We have recently presented evidence that the two types of gastric carcinoma are aetiologically different (Janssen *et al.*, 1991). Now we have shown that growth and spread also are different between the intestinal-type and diffuse gastric carcinoma. We feel that we have contributed to the significance of Pekka Laurén's histopathological classification of gastric carcinoma.

### References

DAVIDSON, H. & TOPOREK, J. (1983). *BMDP technical report no 67.* Revised printing. Los Angeles: BMDP statistical software.

DIXON, W.J. (1983). *BMDP statistical software.* Revised printing. Berkeley: University of California Press.

ERSHLER, W.B., STEWART, J.A., HACKER, M.P., MOORE, A.L. & TINDLE, B.H. (1984). B16 murine melanoma and aging: slower growth and longer survival in old mice. *JNCI*, **72**, 161.

ERSHLER, W.B. (1986). Why tumors grow more slowly in old people. *JNCI*, **77**, 837.

GALL, F.P. & HERMANEK, P. (1985). New aspects in the surgical treatment of gastric carcinoma – a comparative study of 1636 patients operated on between 1969 and 1982. *Eur. J. Surg. Oncol.*, **11**, 219.

HANAI, A., FUJIMOTO, I. & TANIGUCHI, H. (1982). Trends of stomach cancer incidence and histological types in Osaka. Magnus, K. (ed.). *Trends in Cancer Incidence.* Washington; Hemisphere. 143.

HEBERER, G., TEICHMANN, R.K., KRÄMLING, H.-J. & GÜNTHER, B. (1988). Results of gastric resection for carcinoma of the stomach: The European experience. *World J. Surg.*, **12**, 374.

HERMANEK, P. (1986). Prognostic factors in stomach cancer surgery. *Eur. J. Surg. Oncol.*, **12**, 241.

HERMANEK, P. & SOBIN, L.H. (1987). *TNM Classification of Malignant Tumours.* Fourth ed. Springer-Verlag, Berlin, Heidelberg, New York, London, Paris, Tokyo. 43.

INBERG, M., LAURÉN, P. & VIIKARI, S. (1965). Factors influencing survival after radical operation for gastric cancer. *J. Internatl Coll. Surg.*, **44**, 682.

JANSSEN, C.W. Jr, MAARTMANN-MOE, H. LIE, R.T. & MATRE, R. (1991). The age and sex distribution of the intestinal-type and diffuse gastric carcinoma. *APMIS*, **99**, 78.

LAURÉN, P. (1965). The two histological main types of gastric carcinoma: diffuse and so-called intestinal-type carcinoma. *Acta Path. Microbiol. Scand.*, **64**, 31.

MECKLIN, J.-P., NORDLING, S. & SAARIO, I. (1988). Carcinoma of the stomach and its heredity in young patients. *Scand. J. Gastroent.*, **23**, 307.

NODA, S., SOEJIMA, K. & INOKUCHI, K. (1980). Clinico-pathological analysis of the intestinal type and diffuse type of gastric carcinoma. *Jap. J. Surg.*, **10**, 277.

OVERALL, J.E. & SPIEGEL, D.K. (1969). Concerning last squares analysis of experimental data. *Psychol. Bull.*, **72**, 311.

PREHN, R.T. & PREHN, L.M. (1989). The flip side of tumor immunity. *Arch. Surg.*, **124**, 102.

RIBEIRO, M.M., SARMENTO, J.A., SIMOES, S. & BASTOS, J. (1981). Prognostic significance of Lauren and Ming classifications and other pathologic parameters in gastric carcinoma. *Cancer*, **47**, 780.

ROCKWELL, S. (1981). Effect of host age on the transplantation, growth, and radiation response of EMT6 tumors. *Cancer Res.*, **41**, 527.

STEMMERMANN, G.N. & BROWN, C. (1974). A survival study of intestinal and diffuse types of gastric carcinoma. *Cancer*, **33**, 1190.

VISTE, A., EIDE, G.E., HALVORSEN, K., MAARTMANN-MOE, H. & SØREIDE, O. (1986). Prognostic value of Lauren's histopathological classification system and ABO blood groups in patients with stomach carcinoma. *Eur. J. Surg. Oncol.*, **12**, 135.

YUHAS, J.M., PAZMINO, N.H., PROCTOR, J.O. & TOYA, R.E. (1974). A direct relationship between immune competence and the subcutaneous growth rate of a malignant murine lung tumor. *Cancer Res.*, **34**, 722.